# Using data-driven algorithms for semi-automated geomorphological mapping

**DOI:** 10.1007/s00477-021-02062-5

**Published:** 2021-07-30

**Authors:** Elisa Giaccone, Fabio Oriani, Marj Tonini, Christophe Lambiel, Grégoire Mariéthoz

**Affiliations:** Institute of Earth Surface Dynamics, University of Lausanne, Lausanne, Switzerland

**Keywords:** Supervised classification, Direct sampling, Random forest, Geomorphology, Alpine environment

## Abstract

In this paper, we compare the performance of two data-driven algorithms to deal with an automatic classification problem in geomorphology: Direct Sampling (DS) and Random Forest (RF). The main goal is to provide a semi-automated procedure for the geomorphological mapping of alpine environments, using a manually mapped zone as training dataset and predictor variables to infer the classification of a target zone. The applicability of DS to geomorphological classification was never investigated before. Instead, RF based classification has already been applied in few studies, but only with a limited number of geomorphological classes. The outcomes of both approaches are validated by comparing the eight detected classes with a geomorphological map elaborated on the field and considered as ground truth. Both DS and RF give satisfactory results and provide similar performances in term of accuracy and Cohen’s Kappa values. The map obtained with RF presents a noisier spatial distribution of classes than when using DS, because DS takes into account the spatial dependence of the different classes. Results suggest that DS and RF are both suitable techniques for the semi-automated geomorphological mapping in alpine environments at regional scale, opening the way for further improvements.

## Introduction

Classical geomorphological maps are usually obtained by manual mapping and digitization of features from field observations or from topographic data, orthoimagery and remote sensing imagery (Dent and Young 1981; Pain 1985; Mantovani et al. 1996; Batten 2001; Bocco et al. 2001; Lambiel and Pieracci 2008; Lambiel et al. 2016, 2020; Reddy 2018). These approaches are time-consuming, particularly for large areas with limited accessibility, and they are therefore only used for restricted area (Adediran et al. 2004; Schneevoigt et al. 2008). In the last decades, different supervised and unsupervised numerical approaches were proposed to automatically classify key landforms (Smith et al. 2011). In the case of supervised methods, training areas selected with geomorphological expertise are employed (Brown et al. 1998). For unsupervised approaches, algorithms identify the land surface parameters through combinations of predictor variables (Pike 1988). Generally, predictors are morphometric factors derived from digital elevation models (DEMs), such as slope and aspect, and non-morphometric variables that inform vegetation, land cover, lithology and soil (Irvin et al. 1997; Adediran et al. 2004; Gharari et al. 2011). Usually these techniques do not consider the spatial patterns and relations among variables, and may generate misclassifications on terrain discontinuities (Minár and Evans 2008; van Niekerk 2010).

In recent years, more advanced mapping techniques based on machine learning and geostatistics have been developed. These exhibit improved classification performance, especially when based on the analysis of increasingly available high-resolution terrestrial images. In addition, they allow incorporating spatial dependence between multiple locations (Evans 2012; Vannametee et al. 2014).

Developing automatic procedures for geomorphological mapping is relevant in several domains of environmental modelling. For instance, predictive ecological models are used to estimate small-scale species distributions, based on factors influencing vegetation, especially under the effect of global warming (Beniston et al. 2018). Indeed, plant development and distribution depend on indirect variables (e.g. lithology, topography, climate), on direct variables (e.g. nutrients, soil, temperature controls and photosynthetically active radiation), on biotic interactions and disturbances, and on land use (Guisan and Zimmermann 2000; Mod et al. 2016). Using a geomorphological dataset, providing detailed information about processes and landforms, as well as physical disturbances, can improve the predictions of species distribution in mountain environment. Indeed it has been shown that landform morphodynamics is an important factor for alpine plant distribution (Cannone and Gerdol 2003; le Roux and Luoto 2014; Giaccone et al. 2019).

To test the potential of latest-generation supervised classification techniques on geomorphological mapping, we provide here a comparative study on two data-driven algorithms: Direct Sampling and Random Forest. The main goal of this comparative exercise is to perform a semi-automated geomorphological mapping (SAGM) of an alpine environment, and to assess it against an existing geomorphological map elaborated on the field and considered as ground truth.

The first classification method considered is Direct Sampling (DS). DS has recently been employed in different studies, such as for generating stochastic sand channels in aquifer modeling (Huang et al. 2013), gap-filling of daily streamflow time series (Dembélé et al. 2019), simulating rainfall time-series (Oriani et al. 2018), colorizing grayscale or multispectral images (Gravey et al. 2019), or mineral resource estimation (Dagasan et al. 2019). However, despite its ability to account for the spatial dependence of classes, its applicability to geomorphological classification has never been investigated before.

The second approach tested is Random Forest (RF) (Breiman, 2001). RF is widely used in different scientific domains, such as ecology (Prasad et al. 2006; Cutler et al. 2007), permafrost modeling (Deluigi et al. 2017), susceptibility mapping (Stumpf and Kerle 2011; Catani et al. 2013; Leuenberger et al. 2018; Tonini et al. 2020), remote sensing (Chan and Paelinckx 2008; Belgiu and Drăguţ 2016), and also for geomorphological classification (Marmion et al. 2008; Stumpf and Kerle 2011; Veronesi and Hurni 2014). In previous geomorphological applications, the classification was limited to specific landforms belonging to the same morphogenic class (e.g. periglacial landforms, landslides or shaded relief landforms), without considering contiguous areas. Therefore, a general framework including the use of RF for the SAGM, aimed at the accurate depiction of complex landforms in alpine environment, is still lacking.

The DS and RF approaches implemented in the present study seek to provide solutions for a multi-class SAGM. To reach this goal, we tested both algorithms in an alpine area where a classical geomorphological existing map is used for validation.

## Material and methods

### Direct sampling

DS is part of the multiple-point geostatistics (MPS) family of techniques (Mariethoz et al. 2010; Mariethoz and Caers 2014), which simulate a random variable at unknown locations by generating data patterns similar to the ones observed in a given training image (TI) (Strebelle 2002; Caers and Zhang 2004; de Vries et al. 2008; Vannametee et al. 2014). A TI can be a real dataset or a conceptual image of the expected spatial heterogeneity based on prior information (Meerschman et al. 2013). In their pioneer study, Vannametee et al. (2014) showed the applicability of MPS to map 8 landform classes in the French Alps, using the pioneer MPS algorithm SNESIM (Strebelle 2002). With respect to early MPS algorithms, DS can consider both continuous and categorical variables at the same time, which allows using different types of predictor variables.

The DS algorithm generates a random variable on a simulation grid (SG), representing the study zone, by resampling the TI under pattern-matching constraints and calculating the distance $D(\overrightarrow{d}\left(X\right), \overrightarrow{d}\left(y\right))$, i.e. the measure of dissimilarity, between two data events (for more details see Oriani et al. 2021).

1 $$D\left( {\vec{d}\left( X \right), \vec{d}\left( y \right)} \right) = N^{ - 1} \mathop \sum \limits_{n = 1}^{N} {\mathbb{I}}_{{d_{n} \left( x \right) \ne d_{n} \left( y \right)}}$$

where ${d}_{i}\left(\cdot \right)$ is the *n*th datum which composes the conditioning pattern.

In the present case, a categorical variable denoting geomorphological classes is the target variable, manually defined for the TI by geomorphological expertise. A series of morphometric, physical, and remotely sensed variables, defined for both the TI and SG, are provided as predictors for the geomorphological classes. The algorithm identifies correspondences between patterns of these variables, then it sequentially imports in the SG the target variable values (i.e. the geomorphological classes) associated with the most similar patterns found in the TI.

Since DS is a geostatistical simulation algorithm, it does not produce a unique classification, but a (possibly infinite) number of equiprobable scenarios of classes, called realizations. The most probable estimation of the classes can be performed by computing the mode of the realizations (i.e. the most frequent class across all the realizations). In addition, the variability between realizations can be analyzed to estimate the classification uncertainty.

The following DS parameters have to be defined: (1) the maximum fraction of the TI to be scanned *F* [0, 1]; (2) a neighborhood including the number of neighbor pixels to each target; (3) the distance threshold position *T* [0, 1], used to stop or continue the sampling processes if a data event is found in the TI; (4) the number of realizations; (5) the weight for the conditioning data *W* [0, 1]. In our case, we decided to completely scan the TI (*F* = 1) to have access to all the patterns in the training image, with a neighborhood defined as the 9 closest pixels for each predictor, except for the geomorphology variables for which we do not consider spatial neighbors. In the simulation, patterns are compared with a rotation-invariant distance to increase the matching possibilities (Mariethoz and Kelly 2011). The threshold position *T* was set to 0.01 in agreement with Meerschman et al. (2013); 100 realizations were generated and all the variables were given the same weight (*W* = 1).

### Random forest

RF is an ensemble-learning algorithm for classification and regression based on decision trees (Breiman 2001). As a common characteristic of machine learning based approaches, RF is capable of learning from and makes predictions on data, modeling the hidden relationships between a set of input and output variables. Decision trees are supervised classifiers providing decisions at multiple levels and are constituted by root nodes and child nodes. At each node, decisions are taken based on training predictor variables. The number of generated trees (*ntree*) and the number of variables randomly sampled as candidates at each split (*mtry*) are the only parameters that need to be specified by the user. The algorithm then generates *ntree* subsets of the training dataset (counting about one-third of the observations) by bootstrapping (i.e. random sampling with replacement). For each subset, a decision tree is generated and, at each split, the algorithm selects randomly *mtry* variables and computes the Gini index to select the best variable. This step is iterated until each node contains only one or less than a pre-fixed number of data points. The prediction of a new data point is finally computed by taking the average value of all decision trees for regression and the maximum voting for classification, which is the case in the present study. The parameters of the model have been optimized by evaluating the prediction error on those observations that were not used in the training subsets (called “out-of-bag” – OOB). Values were set to 500 for *ntree* and 4 for *mtry,* following a trial and error process. Finally, the relative importance of each variable was assessed by evaluating the mean decrease accuracy, computed by measuring how much the tree nodes using that variable enable reducing the mean square errors estimated with the out-of-bag, across all the trees in the forest.

RF was run twice: firstly with the same input dataset (extracted from the TI) as the one used for DS, and secondly with a balanced dataset. This strategy was adopted because the geomorphological classes are not equally represented in terms of number of pixels per class. We used the SMOTE (Synthetic Minority Over-sampling Technique) function (Chawla et al. 2002), which allows balancing the dataset by artificially generating new examples of the minority classes and by under-sampling the examples of the majority class. The level of over-sampling of the minority classes (*perc.over)* and of under-sampling of the majority classes (*perc.under)* need to be set up by the user, as well as the number of nearest neighbors (*k*) used to generate the new examples of the minority class. In our case, based on trial and error process, we set them as: *perc.over* = 900, *perc.under* = 900 and *k* = 5. In both the runs, unbalanced and balanced, RF was trained on the TI and results predicted on the SG. This selection of the training and the testing dataset (i.e. corresponding to the TI and SG respectively) allowed comparing RF and DS in identical conditions.

Analyses were performed using R free software (R Core Team 2019). Specifically, the packages *randomForest* was employed for the classification procedure and the package DMwR to balance the input dataset (with the function SMOTE).

### Study area and experimental design

**Fig. 1:**
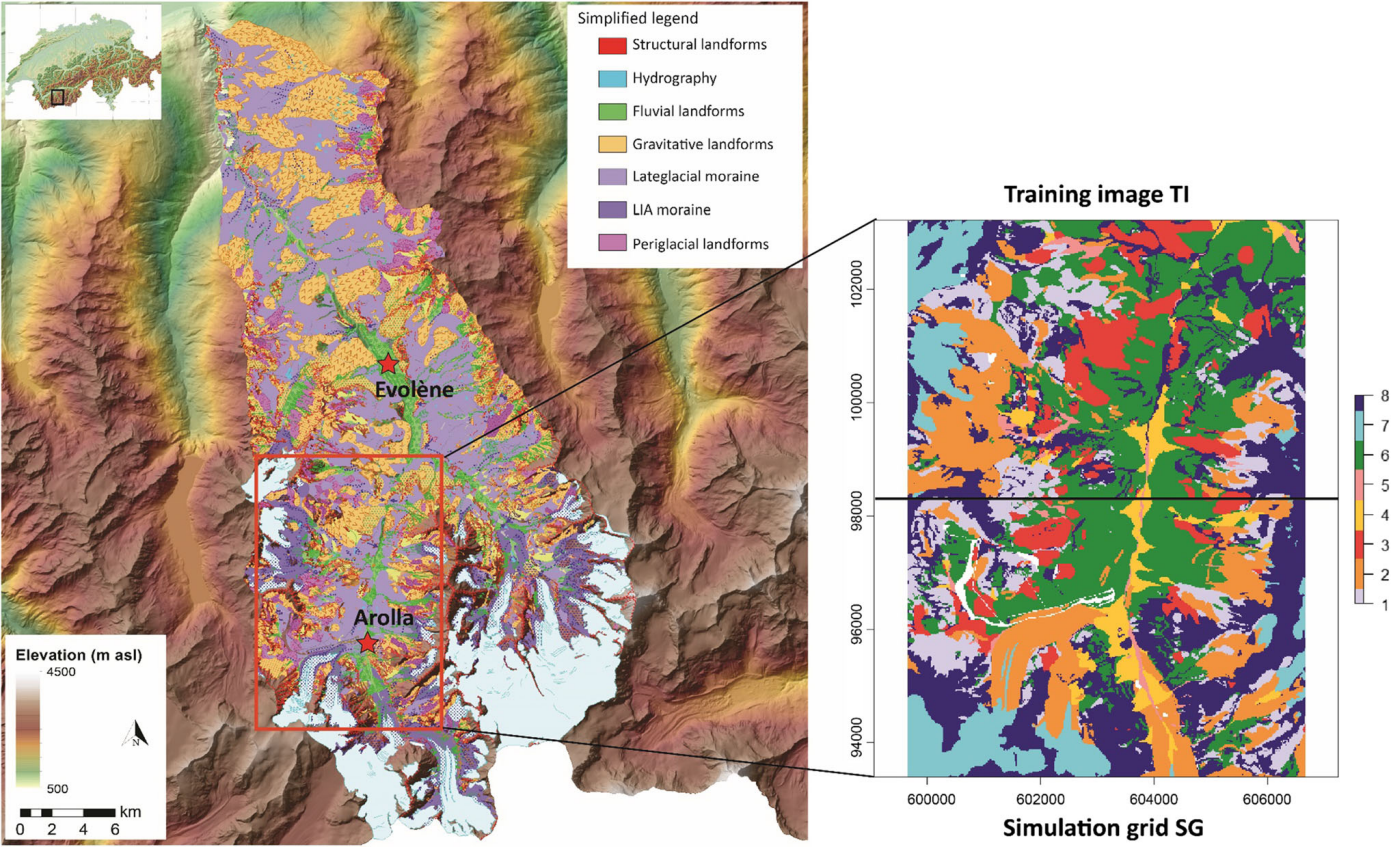
Test area with the geomorphological map elaborated by Lambiel et al. (2016) (left side). On the right side, the area selected for calibrating and running the algorithms to produce the SAGM. The area was divided in two part, the training image (upper part) and the simulation grid (lower part). Legend of the selected area: (1) talus slope; (2) active-inactive rock glacier, debris-covered glacier, Little Ice Age moraine deposit; (3) rockslide, landslide, relict rock glacier; (4) alluvial fan; (5) alluvial plain; (6) Lateglacial deposit; (7) glacier and permanent snow; (8) rock outcrop, rock wall. White zones are excluded from all the calculations. Datum: CH1903 / LV03

The study area corresponds to a rectangular domain of 70 km^2^ in the Arolla valley, located in the southwest Swiss Alps (46° 01’ N, 7° 28’ E) (Fig. 1). We selected this area because a classical geomorphological map is already available and can be used for validation (Lambiel et al. 2016). This map was elaborated using the geomorphological legend established by the University of Lausanne (Schoeneich 1993) and employed in several cases (e.g. Ondicol 2009). It highlights the process categories, the morphogenesis of the landforms and their activity rate. The selected rectangular domain was divided in two equal areas: one used for training (TI) and the second for simulation/testing (SG) the two data-driven algorithms (DS and RF).

Arolla valley is located in the upper part of the Hérens valley, a south-north catchment on the orographic left side of the Rhone River, ranging from 470 to 4357 m a.s.l. Geologically, this valley consists of oceanic sediments and orthogneisses, metagabbros and breccias (Steck et al. 2001). According to the Köppen-Geiger climate classification (Peel et al. 2007), the climate is considered ET (tundra climate) with a mean annual precipitation of 736 mm recorded at the Evolène-Villa weather station (1825 m a.s.l.) for the norm period 1981–2010. The 0 °C isotherm is around 2600 m a.s.l.

Arolla valley is characterized by the presence of several glaciers retreating since the end of the Little Ice Age (nineteenth century), large moraines, widespread periglacial landforms (e.g. active and relict rock glaciers, solifluction lobes), talus slopes and associated debris flows landforms (gullies, fans–Lambiel 2021).

**Fig. 2:**
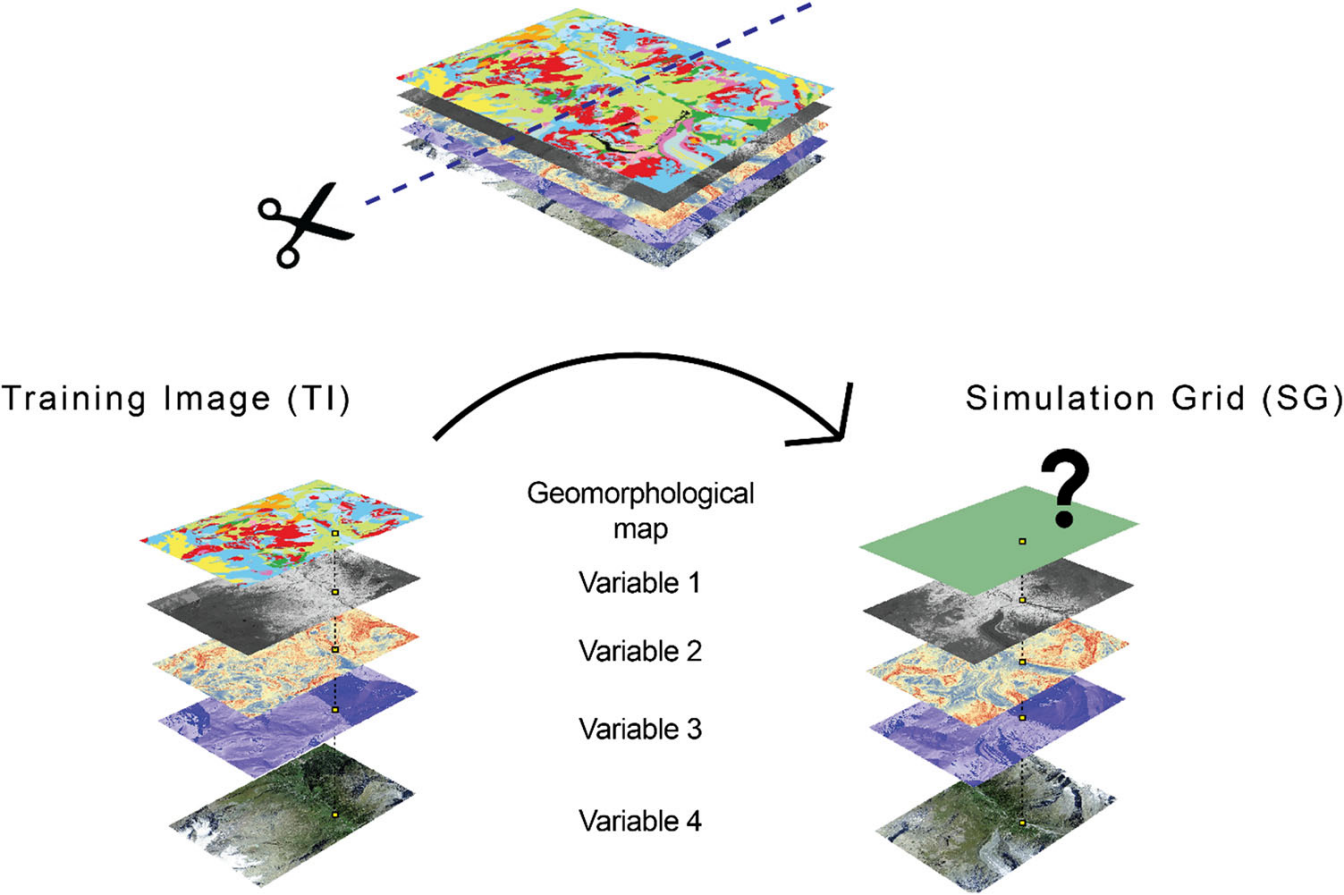
Conceptual model of the test design. The study area is split in two parts, the training image (TI) and the Simulation Grid (SG). Both are composed of the same number of variables with equal resolution grid

The dataset is composed of 13 variables (Table 1): the geomorphological classes, representing the target variable, and 12 predictor variables, including topographical and remote-sensing indicators. The geomorphological classes are informed in the TI. Conversely, in the SG the target variable is uninformed and simulated by the classification algorithms (Fig. 2).

**Table 1:** Variables in the dataset. The orthomosaics and the original DEM were provided by from the Swiss Office of Topography

Variable	Name in dataset	Source
Geomorphology	Geomorphology	Lambiel et al., 2016
R band	Ortho1	aerial orthophoto mosaic (year 2013)
G band	Ortho2	aerial orthophoto mosaic (year 2013)
B band	Ortho3	aerial orthophoto mosaic (year 2013)
Slope	Slope	Alti3D DEM (year 2016)
Sine aspect	Aspect_sin	Alti3D DEM (year 2016)
Cosine aspect	Aspect_cos	Alti3D DEM (year 2016)
Normal curvature	Curvature	Alti3D DEM (year 2016)
Plan curvature	Plan_curv	Alti3D DEM (year 2016)
Profile curvature	Prof_curv	Alti3D DEM (year 2016)
Solar radiation	Solradiation	Alti3D DEM (year 2016)
Flow accumulation	Flow_accumulation	Alti3D DEM (year 2016)
Roughness	Roughness	Alti3D DEM (year 2016)

All the variables were processed under a GIS environment (ArcMap 10.7) and resized on a regular grid with a spatial resolution of 20 m. Flow accumulation and roughness were computed using the TopoToolbox implemented in Matlab (Schwanghart and Kuhn 2010; Schwanghart and Scherler 2014). The aspect was transformed from degrees to sine (aspect_sin) and cosine (aspect_cos) to highlight all cardinal points.

### Geomorphological classification

The original geomorphological map was organized in 11 main classes, grouping more than 100 types of landforms. In the study area, the classes karstic, lacustrine and organic were highly underrepresented and too scarce to be detected by a data-driven classification algorithm. Thus, pixels of these three classes were aggregated with the neighboring pixel of the eight main process-based classes. The anthropic class was excluded from the analysis. The final classification, based on the geomorphological interpretation of the original map performed by the authors, is shown in Table 2.

**Table 2:** Geomorphological classification

Class	Landform
1	Talus slope
2	Active-inactive rock glacier, debris-covered glacier, Little Ice Age moraine deposit (recent and/or active land forms)
3	Rockslide, landslide, relict rock glacier (chaotic deposits partially covered by vegetation)
4	Alluvial fan
5	Alluvial plain
6	Lateglacial deposit
7	Glacier, permanent snow
8	Rock outcrop, rock wall

### Model validation

The predictions made on the SG and resulting from the implemented models were compared with the original geomorphological map (i.e. the observed class) through a confusion matrix (Table 3). This allowed evaluating the performance for each class and computing the overall accuracy and Kappa value (Cohen 1960).

**Table 3:** Confusion matrix

		Predicted class	
		Yes	No
Observed class	Yes	True Positive (TP)	False Negative (FN)
	No	False Positive (FP)	True Negative (TN)

Accuracy is the first evaluation statistic, defined as the ratio of the number of correct predictions over the total predictions:

2 $$Accuracy = \frac{TP + TN}{{TP + TN + FN + FP}}$$

Cohen’s Kappa is a measure of agreement normalized at the baseline of random chance on the dataset:

3 $$k = \frac{{p_{o} - p_{e} }}{{1 - p_{e} }}$$

where *p*_*o*_ is the observed accuracy and *p*_*e*_ is the probability of chance agreement under independence assumption. Kappa values ranges between -1 and + 1, with negative values indicating a complete disagreement among predictions and observations, and positive values an agreement evaluated as slight (0.01–0.20), fair to moderate (0.21–0.60), substantial to almost perfect agreement (0.61–0.80) (Viera and Garrett 2005). The Cohen’s Kappa is generally seen as more informative than the accuracy.

The following evaluation statistics for each class have also been calculated:

4 $$Sensitivity = \frac{TP}{{TP + FN}}$$

5 $$Precision = \frac{TP}{{TP + FP}}$$

Sensitivity measures how often the model correctly assigns a geomorphological class over all the positive observations, and assesses the performance of the model to predict the presence of a geomorphological class when that class is present. Precision is the proportion of geomorphological classes correctly predicted over all the positive predictions.

For a multiclass system, the confusion matrix allows evaluating whether each single class is correctly predicted and to assess the degree of misclassification. This is accomplished by computing the fraction of pixels of class $i$ being labelled as class $j$. Therefore, the matrix diagonal shows the fraction of pixels correctly predicted for each class (corresponding to the sensitivity), while values outside the diagonal represent fractions of misclassified pixels.

In the result section, the DS data with one realization and the mode of 100 realizations are displayed, to highlight how computing the mode improves the results, also from a visual point of view. Furthermore, the probability of each class, computed based on the 100 realizations, is calculated to quantify the precision. For RF, the unbalanced (i.e. the original dataset) and balanced dataset are shown both as categorical values (by taking the maximum vote), and as probabilities (by normalizing the most voted class over the total number of trees).

Experimental variograms (Cressie 2015) were computed for the results of each method to evaluate the degree of spatial dependence of the geomorphological classes. The connectivity index (Hovadik and Larue 2007) was also calculated to estimate the degree of connection of pixels inside the same geomorphological class. Connectivity values range from zero for totally fragmented units, entirely composed by non-adjacent pixels, to one for totally connected units.

## Results

### Direct sampling

**Fig. 3:**
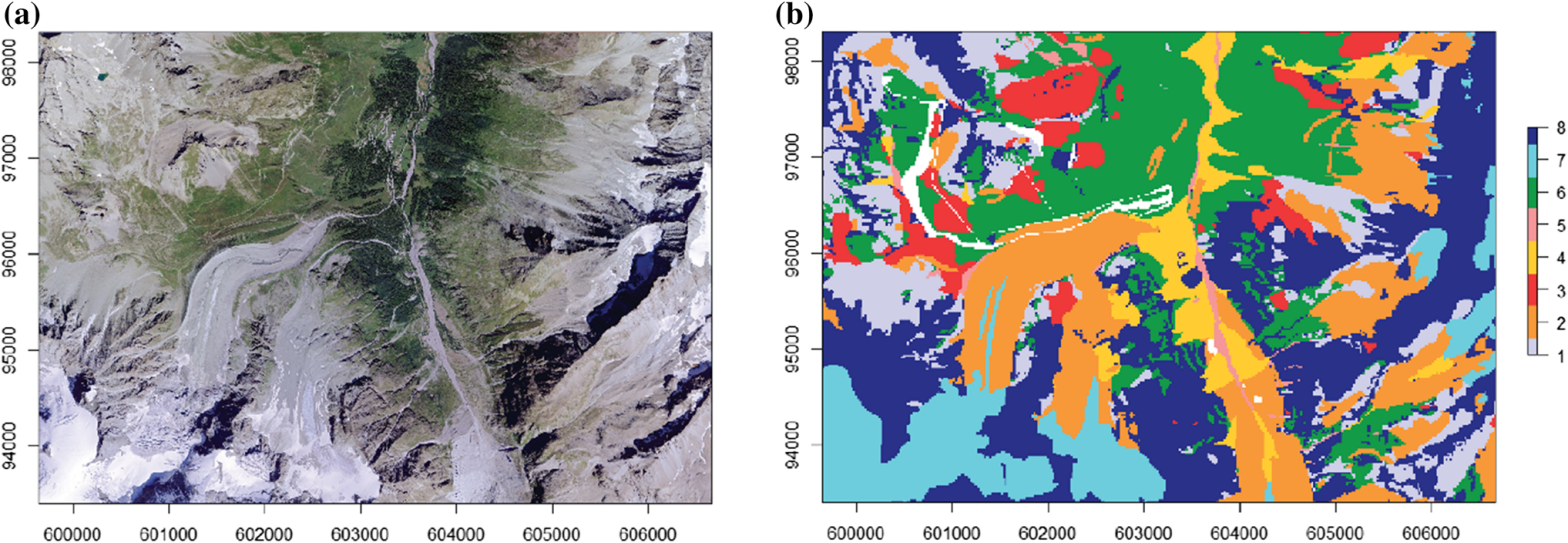
**a** Aerial orthophoto SwissMapRaster © swisstopo (DV084371). **b** Reference geomorphological map for the selected study area. In white, the areas not considered for classification. For the legend, see Fig. 1

**Fig. 4:**
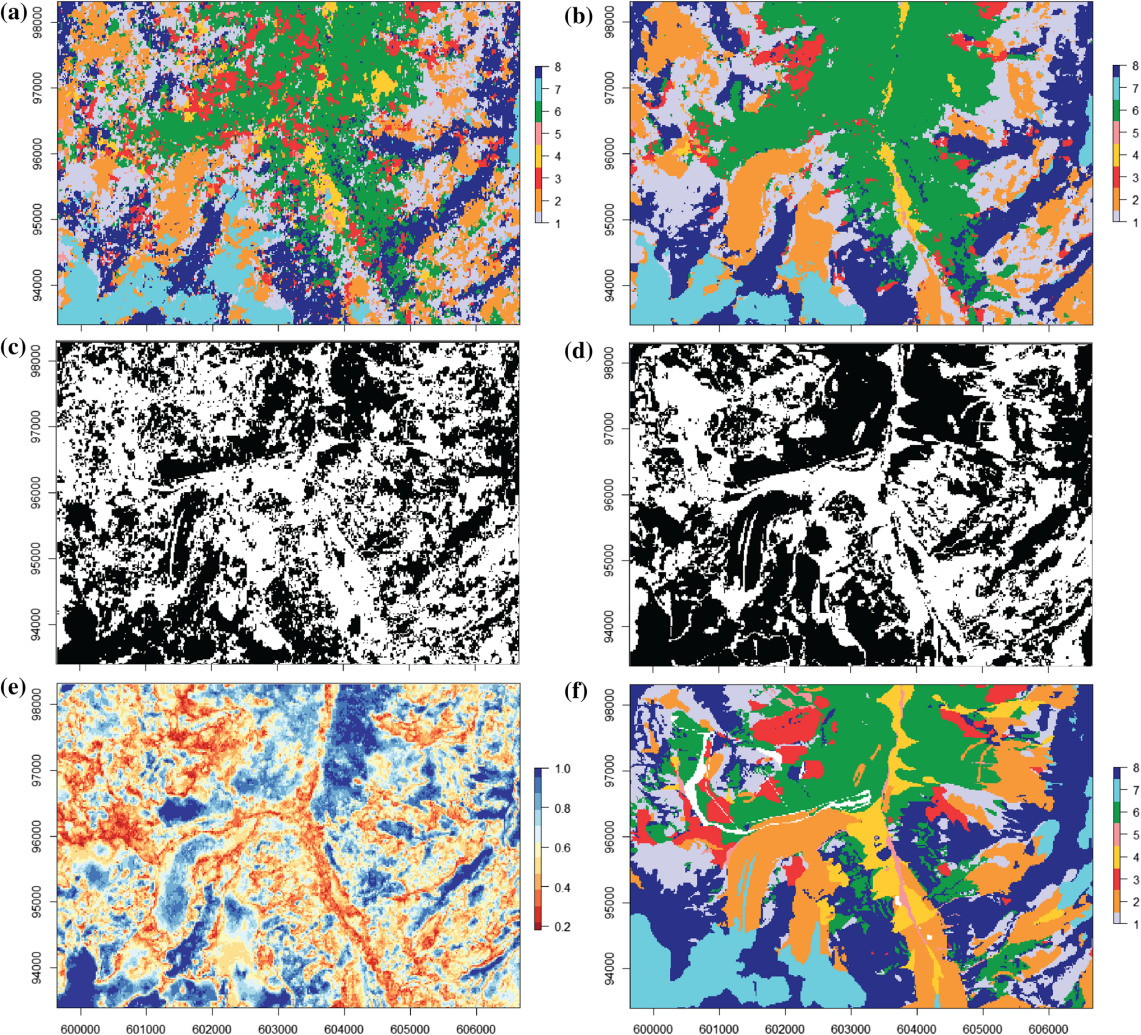
**a** Semi-automated geomorphological map obtained from one DS realization. **b** Semi-automated geomorphological map obtained from the mode of 100 DS realizations. **c** Measure of accuracy of the one DS realization; on black, the pixels correctly simulated, in white, the pixels not corresponding to the reference geomorphological map. **d** Measure of accuracy of the 100 DS simulations. **e** Precision map of the 100 DS realizations, showing the frequency of detection of the class corresponding to the mode. The higher is the probability, the more certain is the estimation. **f** Reference geomorphological map for the selected study area. For the legend, see Fig. 1. In white, the areas not considered for classification

The SAGMs obtained with one DS realization and with the mode of the 100 DS realizations are compared with the aerial orthophoto and the reference map (Fig. 3a and 3b) and are represented in Fig. 4a and 4b. The one-realization results show a low degree of spatial continuity compared to the mode of 100 realizations, but still retains the location of the main patterns. The measure of accuracy of the models is represented in Fig. 4c and 4d as binary values (correctly classified pixels in black, incorrect ones in white). One can identify main landforms such as talus slopes (n° 1), active-inactive rock glaciers, debris-covered glaciers and Little Ice Age moraine deposits (n° 2), Lateglacial deposits (n° 6), glaciers (n° 7), and rock outcrops (n° 8), even if their shapes are clearer in the mode of the 100 DS realizations (Fig. 4b). The class n° 3 (rockslide, landslide, relict rock glacier) presents a structure not coincident with the ground truth. This is also the case for the classes n° 4 (alluvial fans) and n° 5 (alluvial plains), which do not present a coherent pattern with a single realization, but results improved in the mode of 100 realizations.

The measure of precision (Fig. 4e) highlights the areas that were better predicted, with light and dark blue colors (mode-occurrence frequency above 0.7) These areas match mainly with the classes n° 1, 2, 6, 7 and 8, corresponding to Lateglacial deposits in the top-center part, glaciers in the left-bottom part and debris-covered glaciers and their Little Ice Age moraines on the left side, and rock outcrops in the bottom and right side. Instead, in light and dark red colors (mode-occurrence frequency below 0.4), the alluvial fans (n° 4) and the alluvial plains (n° 5) are not correctly simulated in the central part of the area, as well as the rockslides deposits (n° 3) presented mainly in the top-left side.

The confusion matrix (Table 4) confirms the visual interpretation of the results and allows evaluating the predictive power for each class. Concerning the one-realization case (Table. 4a), the best predicted classes are the Lateglacial deposits (n° 6), the glaciers (n° 7) and the rock outcrops (n° 8), attested by high sensitivity values (0.53 for class n° 8 and around 0.6 for classes n°6 and n°7), backed by high precision values (> 0.52). For the other classes, the sensitivity is lower than 0.42; however, the highest values lie on the main diagonal, meaning that the highest fraction of pixels was correctly predicted. The only exception is alluvial fan (n°4) that is more frequently classified as alluvial plain (n°5).

**Table 4:**
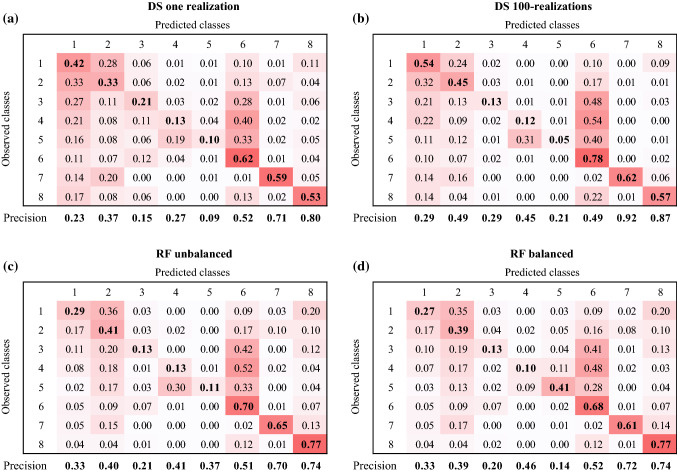
Confusion matrices for: **a** direct sampling (DS) one realization; **b** DS 100 realizations; **c** Random forest (RF) with unbalanced data and **d** RF with balanced data. Values lying on the main diagonal represent the sensitivity

The 100 DS realizations model performs much better than the one-realization case, with highest values of sensitivity for almost all the classes and the class n° 6, 7 and 8 again the best predicted. The class n°4 is still often misclassified (Table 4b).

The overall accuracy for one-realization and for the 100 DS realizations is of 0.46 and 0.55, respectively, with a confidence interval 95% for both, while Kappa is of 0.35 and 0.46, considered a fair (K ∈ [0.21–0.40]) and moderate (K ∈ [0.41–0.60]) agreement (Table 5).

**Table 5:** Summary of the accuracy and Kappa values for all the tested models (DS = Direct Sampling; RF = Random Forest)

	Accuracy	Kappa
DS 1 simulation	0.46	0.35
DS 100 simulations	0.55	0.46
RF unbalanced	0.55	0.44
RF balanced	0.54	0.43

### Random forest

**Fig. 5:**
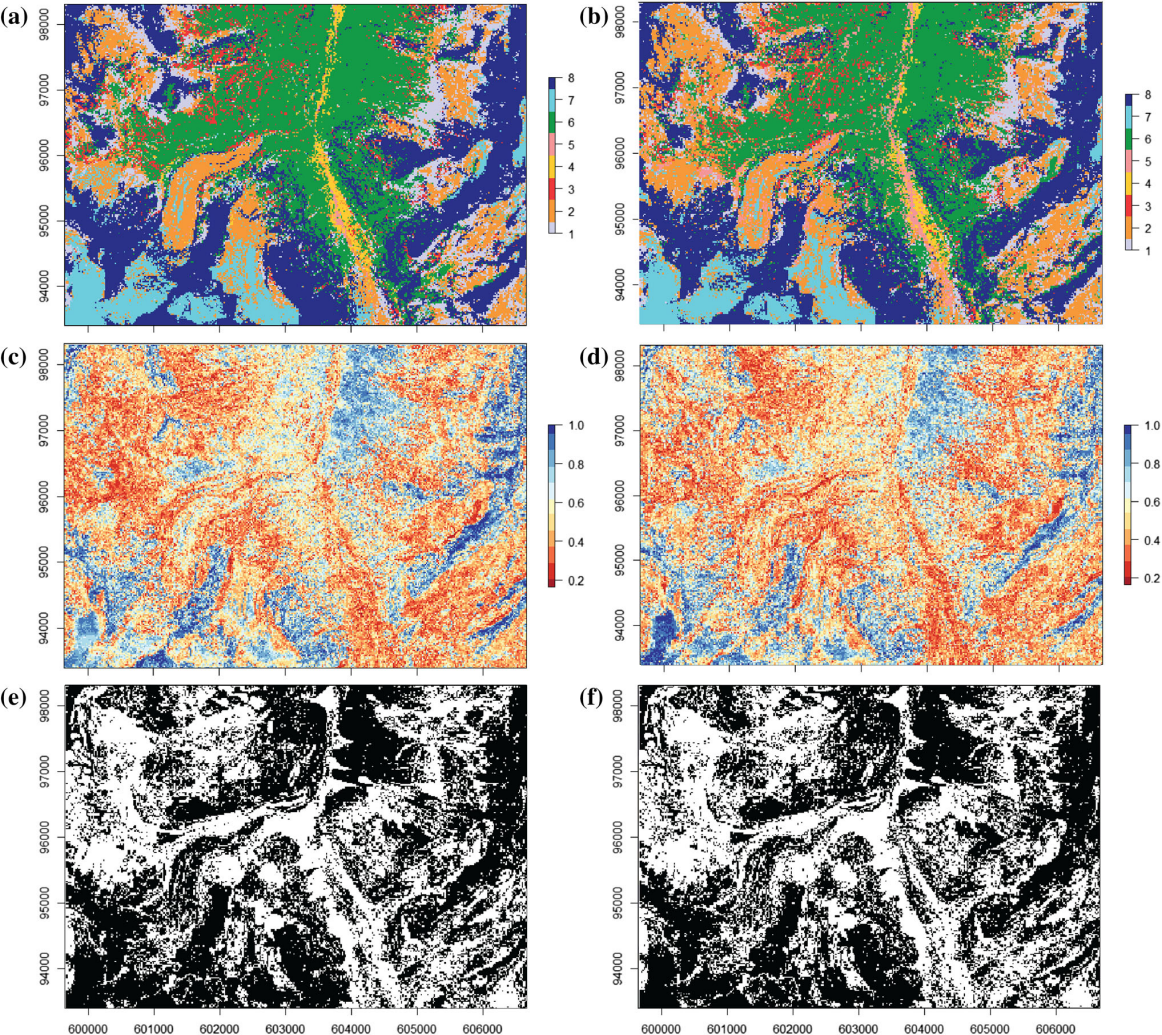
**a** Semi-automated geomorphological map obtained through RF with unbalanced data. For the legend, see Fig. 1. **b** Semi-automated geomorphological map obtained through RF with balanced data. **c** Measure of precision of the RF unbalanced data result. **d** Measure of precision of the RF balanced data result. **e** Measure of accuracy of the RF unbalanced data; on black the pixels correctly classified, and in white the misclassified pixels. **f** Measure of accuracy of the RF balanced data

The classification results from the RF unbalanced and balanced models are shown on Fig. 5a and 5b, respectively. From the visual inspection, the measure of precision (Fig. 5c and 5d) presents high probability values (light–dark blue, > 0.7) only in few areas, such as some rock outcrops in the right and in the left-bottom side of the image, as well as for the glaciers. In the top-central side, the high probability patch indicates a portion of Lateglacial deposits. Even if the different classes are relatively scattered, the class n° 2 (active-inactive rock glaciers, debris-covered glaciers, Little Ice Age moraine deposits) is accurately predicted: in the bottom-left area, for example, two debris covered glaciers are identified with their Little Ice Age moraines. They still present elongated portions of debris-free ice that are efficiently detected. In the right part as well, small glaciers located at the foot of rock walls areas are well identified. Instead, alluvial fans and alluvial plains (classes n° 4 and 5) present low values of precision in the central part of the area where they should be localized, as well the rockslides deposits (n° 3) not correctly simulated especially in the top-left part. The accuracy of the models is represented in Fig. 5e and 5f as binary values (correctly classified pixels in black, incorrect ones in white).

**Fig. 6:**
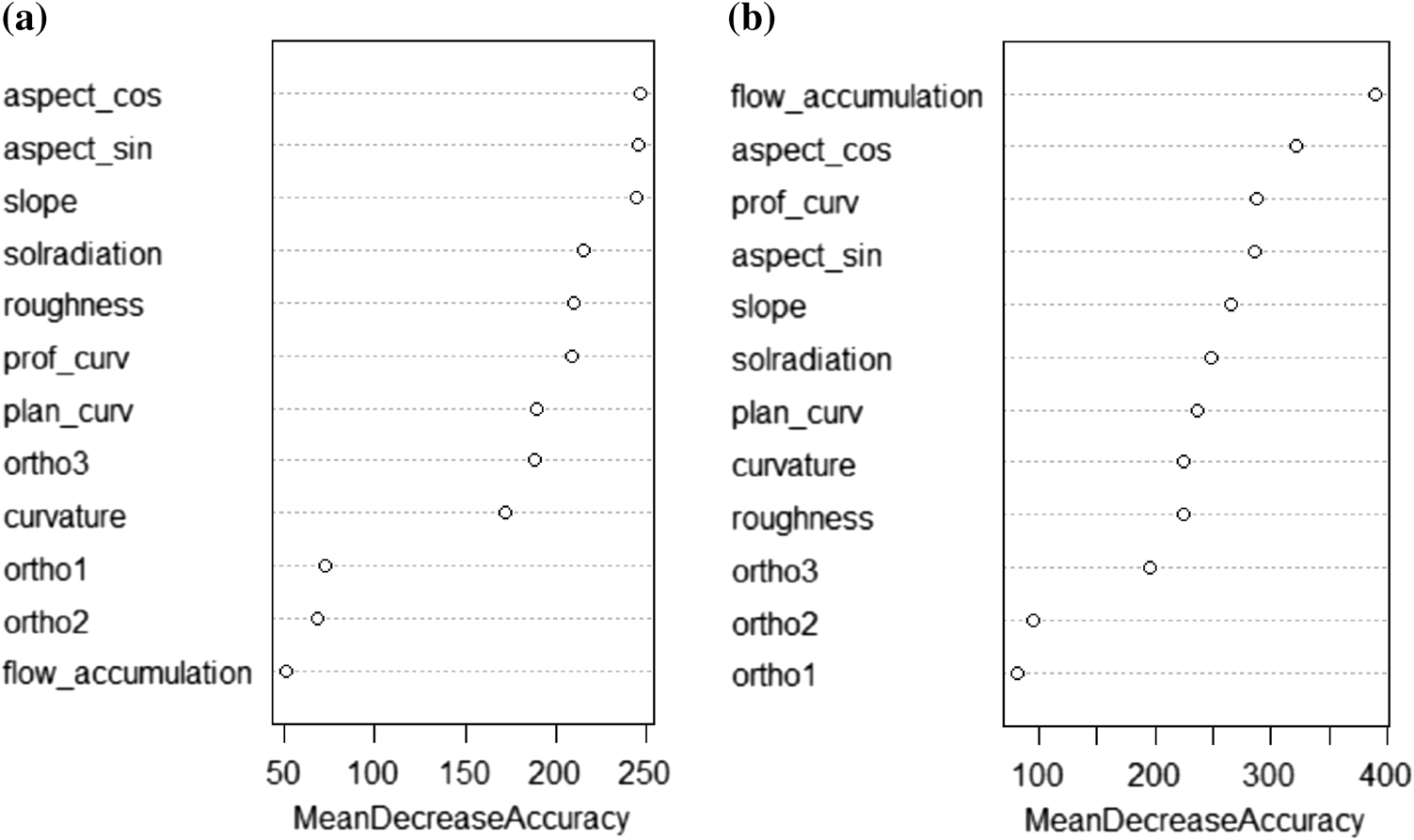
Variable importance ranking as output of the RF estimation. **a** Unbalanced data; **b** Balanced data

**Fig. 7:**
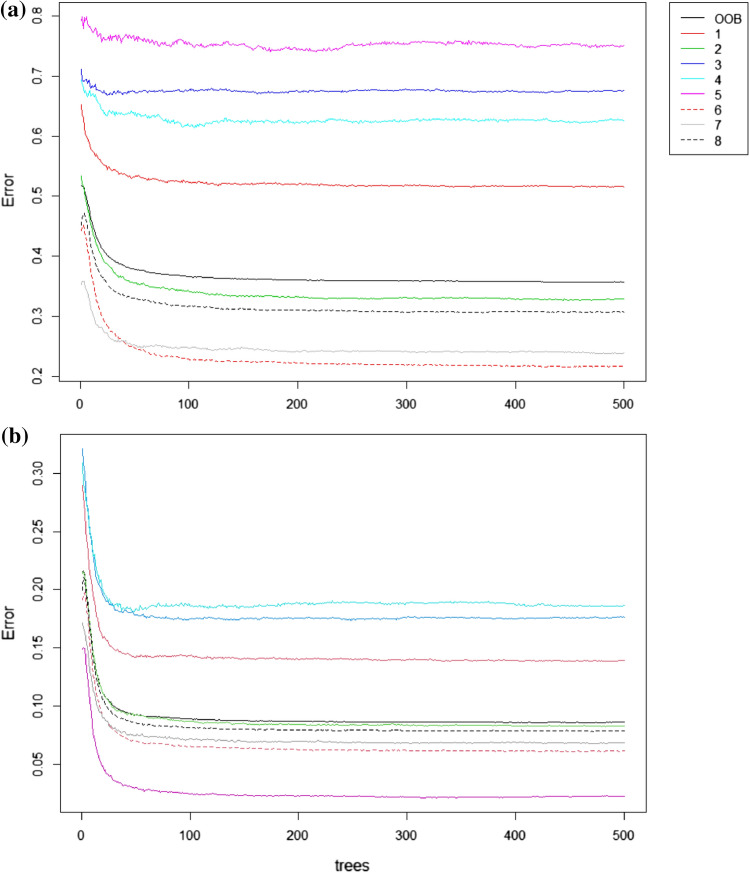
Out-of-bag (OOB) error evolution of RF models trained on the TI. OOB indicates the average error. Numbers from 1 to 8 correspond to geomorphological classes listed in Table 2. **a** Unbalanced data; **b** Balanced data. Legend: 1) talus slope; 2) active-inactive rock glacier, debris-covered glacier, Little Ice Age moraine deposit; 3) rockslide, landslide, relict rock glacier; 4) alluvial fan; 5) alluvial plain; 6) Lateglacial deposit; 7) glacier and permanent snow; 8) rock outcrop, rock wall

The RF algorithm shows a comparable overall performance to DS. According to the mean decrease accuracy, aspect, slope, solar radiation and roughness are key predictors for the unbalanced-data model (Fig. 6a). Flow accumulation, aspect, profile curvature and slope are key predictors in the balanced-data model (Fig. 6b). Other variables offer moderate improvement, except in the case of RGB bands, which have a negligible impact in both cases. Looking at the out-of-bag (OOB) error, plotted as a function of the number of trees, the final value is 35.77% for the unbalanced case (Fig. 7a) and 8.59% for the balanced case (Fig. 7b). It follows that the RF-balanced model allows improving the results only on the same TI, but it does not succeed in generalizing them to the SG. The corresponding minimum values are shown in Table 6. It is clear from these results that the balanced model performs better than the unbalanced one for each class.

**Table 6:** Error for each simulated class, obtained from random forest training on the TI

Class	Unbalanced data	Balanced data
1	0.52	0.14
2	0.33	0.08
3	0.68	0.18
4	0.63	0.19
5	0.75	0.02
6	0.22	0.06
7	0.24	0.07
8	0.31	0.08

The confusion matrices calculated for both models (RF with balanced and̄ unbalanced observations) are shown in Tables 4c and 4d. As for DS, the classes with the best predictions are the Lateglacial deposits (n° 6), the glaciers (n° 7) and the rock outcrops (n°8). For these three classes, sensitivity values are slightly higher that values obtained for DS 100-realizations, with values above 0.61 for both models. In addition, RF-balanced allowed improving the predictability of the classes n°4 (Alluvial fan) and n°5 (Alluvial plain).

The overall accuracy for RF unbalanced and balanced is of 0.55 and 0.54, respectively, with a confidence interval 95% for both, and Kappa values of 0.44 and 0.43, considered a moderate agreement (K ∈ [0.41–0.60]) (Table 5). These statistics are similar to the ones obtained with the DS-100 realization model.

## Discussion

### Geomorphological classification

The first challenge we met in this study was the selection of the appropriate number of geomorphological classes. Indeed, we reduced the classes of the original geomorphological map from 11 main classes, including more than 100 types of landforms, to 8. This was needed due to the complexity of simulating the geomorphological diversity of the landscape, despite recent progresses in machine learning and geostatistical techniques. Many approaches were tested before adopting this combination of geomorphological classes. Whereas Marmion et al. (2008) focused on periglacial landforms in Finland and Stumpf and Kerle (2011) worked on landslides, we aimed at simulating the diversity of alpine landforms trying to preserve, as much as possible, the dominant processes, the shape and the age of the landforms. In this sense, we were inspired by the classification of Vannametee et al. (2014) and Veronesi and Hurni (2014) classifying talus slopes, alluvial fans and rock outcrops, periglacial-glacial active deposits (active-inactive rock glaciers, debris-covered glaciers, Little Ice Age moraine deposits), gravitative and/or inactive deposits (rockslides, landslides, relict rock glaciers), fluvial deposits, Lateglacial deposits and glaciers. Moreover, increasing the number of classes is not appropriate because it reduces the number of pixel occurrences for each class and consequently decreases the classification performance (Vannametee et al. 2014).

### Algorithm accuracy and efficiency

As shown in the results section, the mode calculation on 100 realizations substantially improves the DS results compared to a single realization, therefore we discuss mainly the mode results here. Globally, DS and RF show similar performances. Indeed, the overall accuracy has only one point percentage of difference (~ 0.55), and two/three for the Kappa value, with values in the range of what is considered as a moderate agreement (~ 0.45).

The DS provides more visually appealing results because, after the mode calculation, defined units representing geomorphological features are better delineated. Furthermore, since it takes into account the spatial dependence between neighborhood attributes, the DS classification is less noisy (Vannametee et al. 2014). Preserving the connectivity of the classes can have important effects when the resulting geomorphological maps are used to parametrize hydrological or other physical models.

Computationally RF is about 5–10 times faster than DS, but produces more scattered simulations since the model does not include the spatial dependence. Isolated pixels are generally not desirable for geomorphological mapping, and compact regions are rather sought. This said, RF provides more information on the predictive power of the conditioning variables, which is precious to guide the variable choice.

Despite the encouraging results of RF with balanced data on the TI (OOB mean error of 0.10 vs 0.46 for the RF-unbalanced), the RF performance on the SG is similar to DS. The reason could be the topographical complexity and geomorphological heterogeneity of the area, which makes the SG and TI less homogeneous and similar to each other, and the prediction problem non trivial. Indeed, it is possible that some landforms were underrepresented in the TI compared to the SG or vice versa.

### Performance over different geomorphological units

Lateglacial deposits (n° 6), glaciers (n° 7) and rock outcrops (n° 8) result to be the classes with the highest sensitivity values and the highest precision. Since Lateglacial deposits are generally vegetated, the RGB bands of aerial orthophotos are key factors for the corresponding categories, even if RGB bands are less important variables (Fig. 6). Instead, slope angle is decisive for rock outcrops / rock walls because it allows isolating these landforms with values > 40°. RF is particularly performant in detecting the classes n° 7 and 8, with a sensitivity higher than for DS. Conversely, the sensitivity value of class n° 6 is higher with DS.

Moderate sensitivity values are calculated for talus slopes (n° 1) and for active-inactive rock glaciers, debris-covered glaciers and Little Ice Age moraine deposits (n° 2), but in both cases the DS shows better performance than RF (respectively 0.54 and 0.45 for DS, 0.29 and 0.41 and RF). The reasons can be linked to the morphology of these landforms. Indeed, talus slopes are constituted by debris with a slope angle of 33–40° (Chandler 1973; Francou and Manté 1990), with the size of debris increasing towards the bottom of the slope. On the other hand, active-inactive rock glaciers, debris-covered glaciers and Little Ice Age moraine deposits present a large intra-variability in debris size, slope and shape. Despite these differences, which are clearly recognizable by geomorphologist, both algorithms confuse the two classes, with zones of more difficult interpretation, probably because the predictor variables are not informative enough to make a clear distinction.

The lowest sensitivity values are found for rockslides, landslides and relict rock glaciers (n° 3), alluvial fans (n° 4) and alluvial plains (n° 5). The challenging simulation of alluvial fans was already noted by Veronesi and Hurni (2014). Indeed, the slope angle of this type of landform can vary naturally, but it is also subject to land use changes, especially at the bottom part of the valleys that are under anthropic influence for water management (Gabbud and Lane 2016). This is valid also for alluvial plains, which are subjected to changes in water flow, sediment contribution and human activities (Gabbud et al. 2019). Consequently, they present a highly variable morphology.

Regarding the class n° 2, its intrinsic heterogeneity could be responsible for the low sensitivity. Indeed, rockslides and landslides are characterized by chaotic deposits, with rock fragments, soil, and vegetated portions. Conversely, relict rock glaciers have better defined outlines and are often colonized by vegetation.

**Fig. 8:**
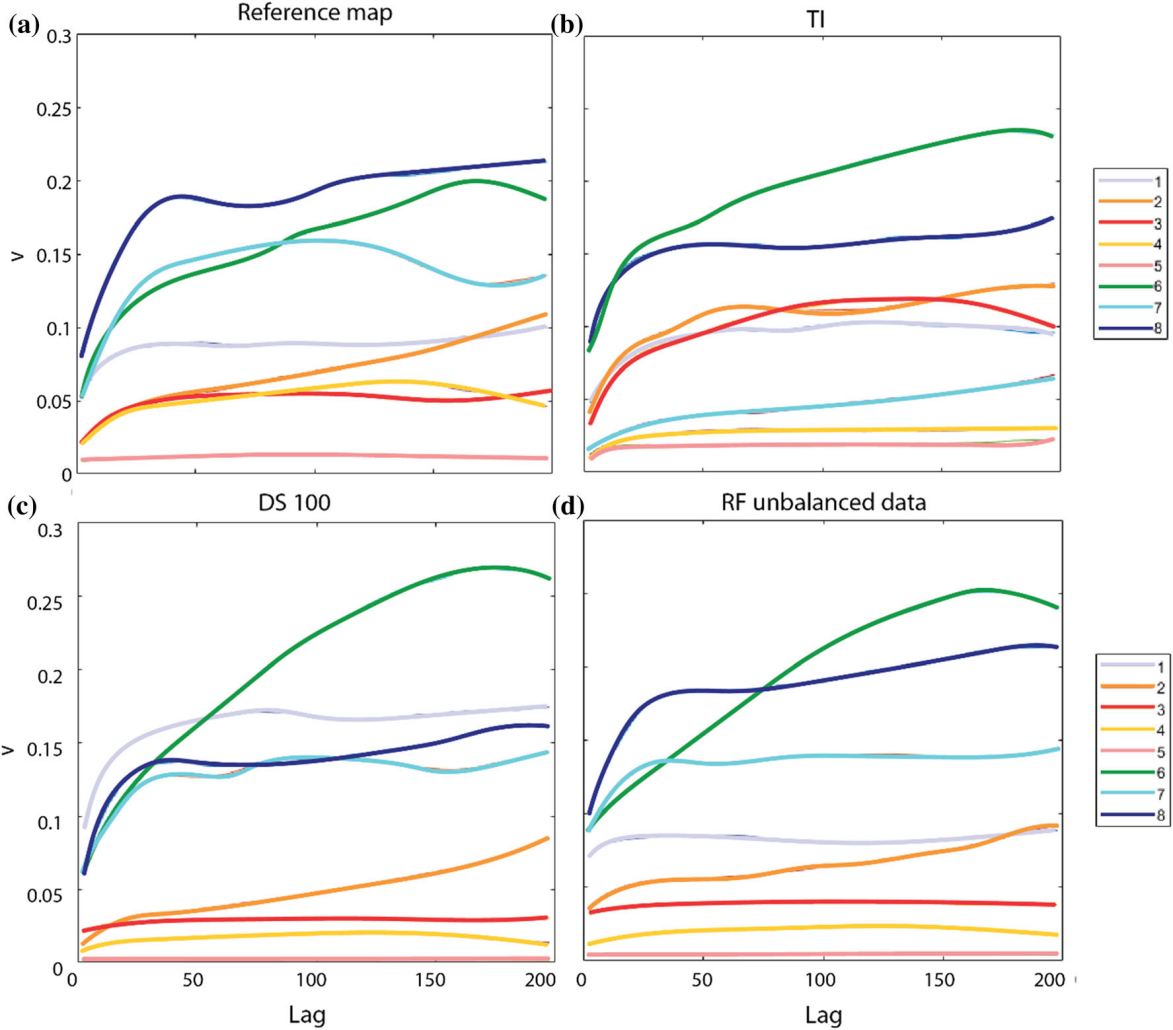
Variograms calculated on the geomorphological reference map (**a**), on the TI (**b**), on DS 100 simulations (**c**) and on RF with unbalanced data (**d**). v: variogram; lag: lag distance between pixels. Legend: 1) talus slope; 2) active-inactive rock glacier, debris-covered glacier, Little Ice Age moraine deposit; 3) rockslide, landslide, relict rock glacier; 4) alluvial fan; 5) alluvial plain; 6) Lateglacial deposit; 7) glacier and permanent snow; 8) rock outcrop, rock wall

**Fig. 9:**
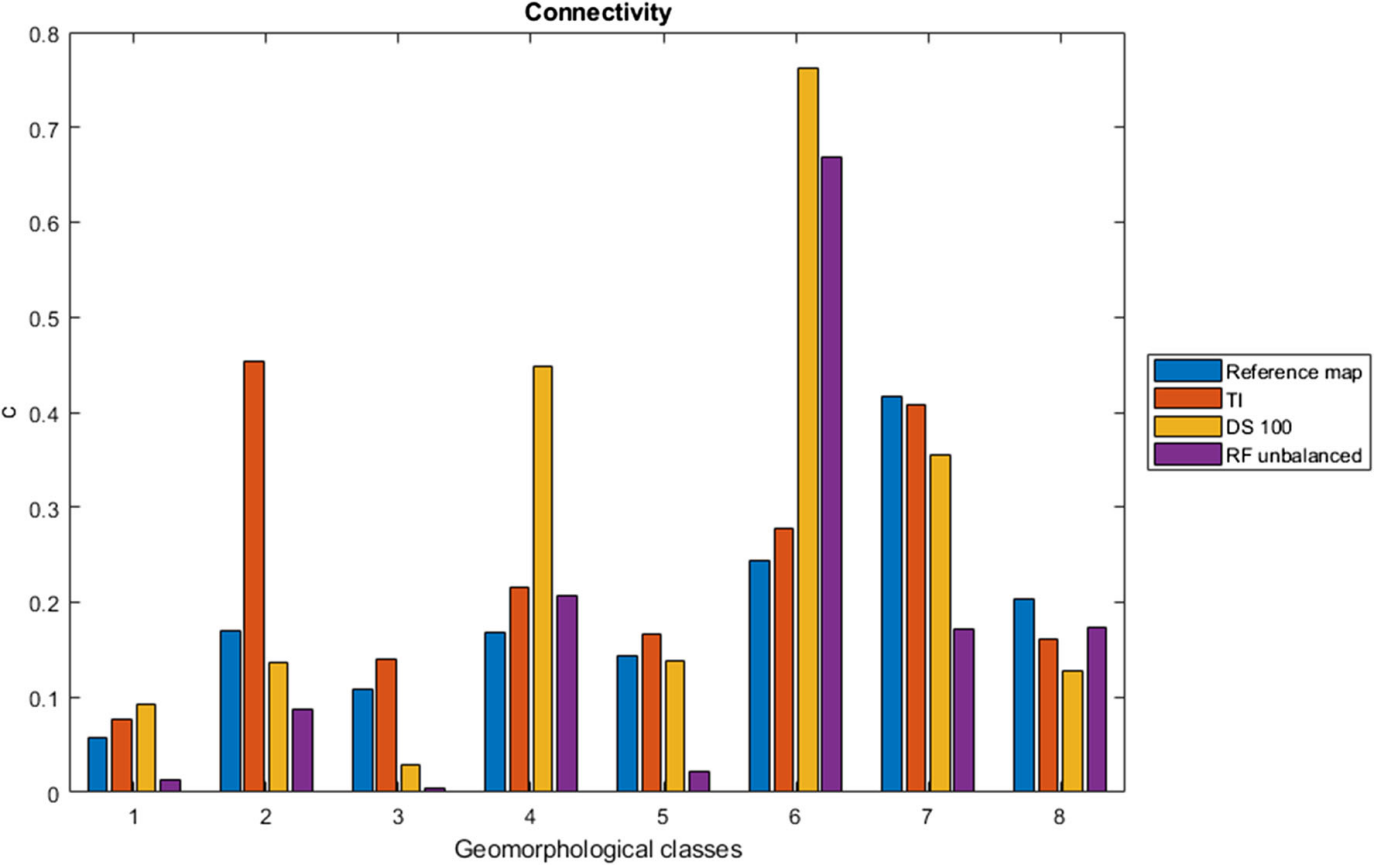
Connectivity calculated for each class for geomorphological reference map, TI, DS 100 simulations and RF with unbalanced data. Legend: (1) talus slope; (2) active-inactive rock glacier, debris-covered glacier, Little Ice Age moraine deposit; (3) rockslide, landslide, relict rock glacier; (4) alluvial fan; (5) alluvial plain; (6) Lateglacial deposit; (7) glacier and permanent snow; (8) rock outcrop, rock wall

The variograms computed for the results of each method to evaluate the degree of spatial dependence of the geomorphological classes (Fig. 8) allow better interpreting these results and putting them in comparison with the reference map (Fig. 8a) and the training image (Fig. 8b). DS (Fig. 8c) overestimates the classes n° 1 and 6 and underestimates classes n° 4, 5 and 8. RF (Fig. 8d) overestimates only the class n° 6 and underestimates classes n° 4 and 5. Variograms of simulated classes n° 2, 3 and 7 have behaviors similar to those of the reference map. However, the connectivity (Fig. 9) shows that DS is slightly more accurate than RF because it maintains for the most part of the classes a connectivity between pixels of the same geomorphological class similar to that of the reference map.

### Predictor variables and training location choice

Another issue we faced in this study was the selection of the predictor variables. As shown by the mean decreasing accuracy (Fig. 6), the simulation is highly sensitive to aspect, slope, solar radiation and profile curvature. Roughness provided also additional information because some landforms have marked surface irregularities. This is the case for rock walls, which present high roughness values compared to other more homogeneous landforms, such as glaciers. Instead, flow accumulation is the most important variable for the model with balanced data, but the less important for the model with unbalanced data. This was not expected because, defining the number of upstream cells based on a flow direction, this variable helps identifying fluvial landforms such as alluvial fan and fluvial deposits.

The choice of the training location is fundamental to obtain valid results (Tuia et al. 2011). Even if there are no strict guidelines regarding the recommended size of a TI, this should contain a sufficient and possibly redundant variety of spatial patterns and a representative distribution of landform classes in order to avoid sampling biases (Caers and Zhang 2004). The spatial resolution must be high enough to correctly represent the spatial distribution of the patches of all classes, without incurring overly long computing times, which can easily increase for DS in case of very large image arrays. It is worth repeating that, to ensure a fair comparison between the two data-driven algorithms, the same TI and SG defined and optimized for DS were used also for RF. In light of the results obtained in the present study, further investigation could consider to implement a balanced-RF procedure using a sub-ensemble of the observations randomly selected over the entire study area to train the model, a second set for validation purpose and finally to predict the results on new data. Also, surface texture and texture indices such as top/bottom hat operators could be used to create new predictor variables that inform the spatial relation between landforms (Aptoula and Lefèvre 2007; Trevisani et al. 2012; Trevisani and Rocca 2015).

Lastly, given their limitations, the semi-automated mapping tools tested in this study are not intended as a substitution of the expert role, but instead as an empowering tool. In particular, the geomorphologist plays a crucial role in the choice and survey of the training areas, which can be multiple and representative of different sub-environments. Moreover, the examination of the output precision maps (Fig. 4e, 5c and 5d), together with the orthophotos, can be used to identify high-uncertainty zones that need in-situ analyses. High uncertainty zones can also indicate the need of expanding the survey of training areas over underrepresented geomorphological units. In this way, the reliability of the obtained mapping product can be significantly improved by the expert knowledge, but with the big advantage of a limited fieldwork with respect to the covered area.

## Conclusion

In this research, we compared two semi-automatic geomorphological mapping (SAGM) methods, the first based on the Direct Sampling (DS) algorithm and the second on Random Forest (RF). The aim was to explore the feasibility of the SAGM at the regional scale, using a pre-classified map to train the methods (training image, TI) and a target study area to test them (simulation grid, SG). To the best of our knowledge, this represents the first application of DS and RF to morphogenetic classification. The classification used twelve environmental predictor variables, including topographical and remote-sensing indicators. A classical geomorphological map was available for the study area and used for training and validation.

Both methods show similar results in terms of accuracy and are deemed appropriate for SAGM, albeit with different trade-offs in terms of spatial smoothness and computational performance. The map elaborated using RF presents a noisier spatial distribution of classes, but gives more insights on the choice of the predictor variables to be used and it is more efficient in terms of computation time compared to DS. This can be attributed to the fact that DS explicitly takes into account the spatial dependence of the different classes. Some classes, such as the Lateglacial deposits, glaciers and rock outcrop areas, resulted in high detection scores, highlighting the suitability of the employed methods for the generation of geomorphological maps in alpine environment. However, other classes such as alluvial fans and alluvial plains were weakly detected, indicating that not all landforms can be appropriately classified with the proposed strategies and algorithm setup, especially if some classes are underrepresented in the TI.

The tested approaches are useful to provide geomorphological maps for vegetation models or other applications and can be employed by the geomorphologist as starting point for additional surveys. Our study identified a potential to use such methods at a regional scale, and also possibly with different geomorphological characteristics than the ones used here. Nevertheless, the geomorphological classification employed in the current analysis can be improved upon. Future researches can be devoted to the optimal choice of the input geomorphological dataset and predictor variables, such as the ones related with surface texture, helping to preserve the spatial relationship between the detected landforms.
